# Bacterial Extracellular Vesicles in the Strategic Interplay Between Pathogens and Hosts

**DOI:** 10.3390/microorganisms14061362

**Published:** 2026-06-18

**Authors:** Jiahui Liang, Mi Li, Jingjing Xu, Shengxia Chen

**Affiliations:** Department of Laboratory Medicine, School of Medicine, Jiangsu University, Zhenjiang 212000, China; q893358108@163.com (J.L.); lmionly@163.com (M.L.); 15861396268@163.com (J.X.)

**Keywords:** bacterial extracellular vesicles, bacteria–host interactions, immune modulation, Gram-negative bacteria, Gram-positive bacteria

## Abstract

Bacterial extracellular vesicles (BEVs) are nanoscale spherical lipid bilayer structures secreted by bacteria, including outer membrane vesicles (OMVs) released by Gram-negative bacteria and membrane vesicles (MVs) produced by Gram-positive bacteria. Although the biogenesis of BEVs requires substantial energy expenditure, these vesicles provide bacteria with strategic advantages in the evolutionary interplay between pathogens and hosts. BEVs contribute to bacterial adaptation to environmental stress by remodeling membrane components, eliminating toxic substances, promoting biofilm formation, and mediating the interbacterial transfer of antibiotic resistance determinants. They can also function as decoys to protect bacteria from bacteriophage or antibiotic attack, deliver virulence factors, modulate host immune responses to facilitate bacterial colonization, and mediate interspecies competition. This review summarizes the central roles of BEVs as bacterial mediators of environmental responses, with particular emphasis on their involvement in immune regulation, environmental adaptation, and interspecies competition, thereby providing new insights into pathogen evolutionary strategies.

## 1. Introduction

The secretion of bacterial extracellular vesicles (BEVs) is a widespread phenomenon among bacteria [1]. However, their biogenesis and evolutionary significance remain associated with a fundamental paradox. From the perspective of energy metabolism, BEV production requires substantial resource investment, and the membrane components and virulence-associated factors carried by these vesicles may activate host immune responses [2]. In contrast, from the perspective of bacterial survival strategies, this process is expected to confer certain advantages that outweigh its biological costs.

This paradox raises two central questions: how bacteria dynamically regulate BEV secretion to balance survival costs and benefits, and how BEVs function as mediators that allow bacteria to gain advantages in host–bacteria interactions. From an evolutionary perspective, all biological behaviors must ultimately provide some form of benefit to the organism. Bacteria, particularly pathogenic bacteria, must continuously cope with hostile host environments, and BEV secretion appears to represent an important strategy for bacterial survival under such adverse conditions. In this review, we examine BEV secretion from the bacterial perspective and discuss whether, and how, this process contributes to bacterial fitness and survival.

## 2. Formation and Structural Composition of BEVs

The study of bacterial extracellular vesicles (BEVs) can be traced back to the 1960s, when soluble lipopolysaccharide (LPS) was detected in cell-free culture supernatants of *Escherichia coli* grown in lysine-limited medium, and vesicle-like structures were observed by electron microscopy. Owing to the technical limitations at that time, these membrane-enclosed nanoparticles were initially regarded as debris derived from dead cell lysis [3,4].

With advances in transmission electron microscopy and ultracentrifugation, it was gradually recognized that outer membrane vesicles (OMVs) secreted by Gram-negative bacteria are not merely by-products of cell death, but rather intrinsic features of the bacterial life cycle. Gram-negative bacteria continuously release OMVs during natural growth, a finding that substantially challenged the prevailing understanding at that time [5].

More remarkably, the long-standing assumption that the thick peptidoglycan cell wall of Gram-positive bacteria prevents vesicle formation was overturned in 1990, when vesicle structures were first observed in *Bacillus cereus* and *Bacillus subtilis* using electron microscopy [6]. Since the beginning of the 21st century, further studies have shown that typical lipid bilayer vesicles with diameters ranging from 20 to 400 nm can be isolated from the culture supernatants of diverse Gram-positive bacteria, ranging from pathogenic *Staphylococcus aureus* to commensal lactic acid bacteria. These findings have confirmed the universality of BEVs as a conserved secretion system in prokaryotes [7].

### 2.1. BEVs Derived from Gram-Negative Bacteria

#### 2.1.1. Composition and Structure of Gram-Negative Bacterial BEVs

**Figure 1:**
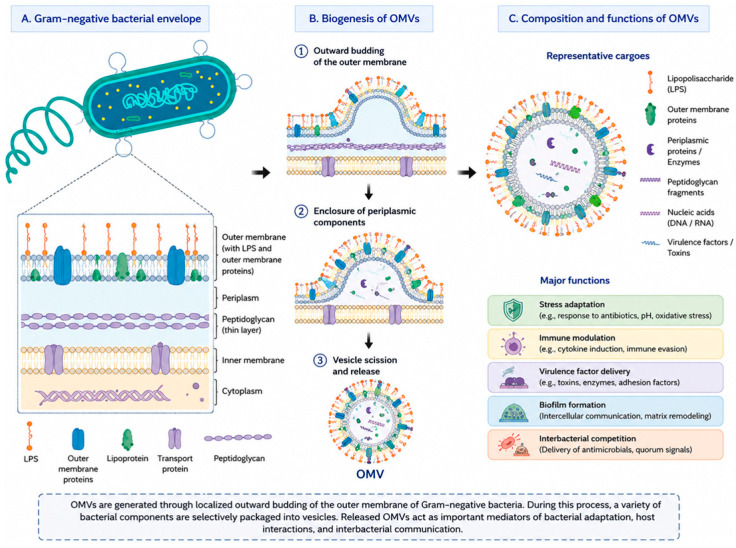
Biogenesis and composition of outer membrane vesicles (OMVs) derived from Gram-negative bacteria. The figure was created using BioGDP.com [8].

The envelope system of Gram-negative bacteria consists of three key structural layers (Figure 1): (1) a phospholipid-rich inner membrane (IM); (2) an outer membrane (OM), primarily composed of lipopolysaccharide (LPS); and (3) the periplasmic space located between the two membranes, which contains a thin peptidoglycan (PG) layer. Although the periplasmic space represents an oxidative environment, it lacks nucleotide-based energy carriers such as ATP and GTP; therefore, material transport across this compartment mainly depends on the proton motive force generated across the inner membrane [5].

Outer membrane vesicles (OMVs) are bilayered membrane structures formed through localized outward bulging of the outer membrane, followed by vesicle detachment. They are typically approximately 20–300 nm in diameter. The lumen of OMVs contains periplasmic components, including soluble proteins, PG fragments, outer membrane-derived integral membrane proteins, and lipid molecules. In addition, OMVs are enriched with various signaling molecules, such as bacterial DNA and quorum-sensing molecules [5,6,9,10].

OMV biogenesis is an active secretory process that occurs during the proliferative phase of bacterial growth. As key mediators of pathogen–host interactions, OMVs are enriched on their surface with diverse pathogen-associated molecular patterns (PAMPs), including endotoxin, peptidoglycan, and bacterial nucleic acids. These molecules can be recognized by host pattern recognition receptors (PRRs), thereby triggering innate immune responses [11,12,13].

#### 2.1.2. Biogenesis of Gram-Negative Bacterial OMVs

Although OMV biogenesis is associated with considerable energy expenditure, multiple lines of evidence support its evolutionary advantage as an adaptive strategy in Gram-negative bacteria. By mediating pathogen–environment interactions, OMVs help regulate the bacterial survival microenvironment. The mechanisms underlying OMV formation can generally be summarized into three theoretical models.

First, OMV formation may result from physical or chemical stress imposed on the outer membrane. When bacteria encounter environmental stress, the accumulation of misfolded proteins, peptidoglycan (PG) fragments, and lipopolysaccharide (LPS) within the periplasmic space can generate local tension, thereby inducing outward bulging of the outer membrane and subsequent vesicle formation. This process not only relieves mechanical stress within the membrane system but also reduces damage to cellular components by encapsulating toxic substances such as reactive oxygen species (ROS) [14,15,16]. Therefore, OMV production may represent a rapid-response mechanism by which bacteria cope with environmental stress. Under these conditions, OMV formation appears to be closely associated with changes in environmental pressure.

Second, OMV production may be closely related to weakened anchoring between the outer membrane and the PG layer. Studies have shown that when the number of proteins linking the outer membrane to the PG layer is reduced, the outer membrane loses mechanical support and undergoes localized protrusion, thereby markedly promoting OMV secretion [11]. In addition, antibiotics and bacterial autolysins can further enhance the tendency of the outer membrane to detach from the PG layer by inhibiting peptidoglycan cross-linking or degrading its network structure. Under such stress conditions, excessive OMV secretion may indirectly enhance bacterial antibiotic resistance by enriching and exporting components such as antibiotic-hydrolyzing enzymes and toxin receptors [9,17,18].

Third, OMV formation is highly dependent on interactions among LPS molecules. When environmental factors disrupt LPS–LPS interactions, local outer membrane fluidity increases, providing a thermodynamic driving force for OMV budding [19].

Collectively, OMV secretion is essentially an efficient stress-adaptation strategy shaped by evolutionary pressure. Although their biogenesis requires substantial energy investment, OMVs provide a critical buffering window for bacterial survival when membrane-toxic substances accumulate or the surrounding environment changes abruptly. This is achieved through the rapid export of harmful components and the remodeling of membrane homeostasis. Such a “high-cost, high-benefit” survival trade-off may explain why OMV secretion has been retained and optimized through natural selection. Under critical conditions that threaten bacterial survival, the net adaptive benefit of OMV production far exceeds its metabolic cost, thereby consolidating OMV biogenesis as a conserved survival program in Gram-negative bacteria.

### 2.2. BEVs Derived from Gram-Positive Bacteria

#### 2.2.1. Composition and Structure of Gram-Positive Bacterial BEVs

BEVs produced by Gram-positive bacteria are commonly referred to as membrane vesicles (MVs) because of their distinct biogenesis pathways, and their diameters generally range from 20 to 500 nm. Most previous studies have focused on OMVs derived from Gram-negative bacteria, resulting in relatively limited reports on MVs produced by Gram-positive bacteria. Nevertheless, Gram-positive bacteria, ranging from pathogenic species to commensal and probiotic bacteria, generally possess the capacity to secrete vesicles [20].

**Figure 2:**
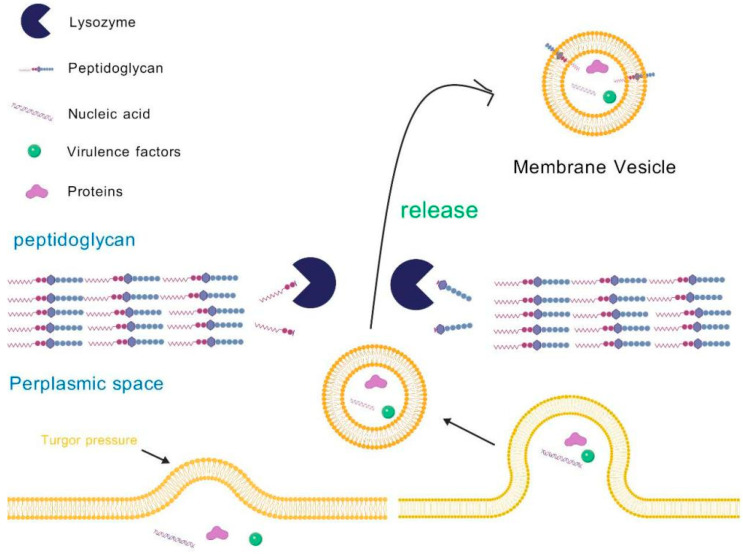
Biogenesis and composition of membrane vesicles (MVs) derived from Gram-positive bacteria. The figure was created using BioGDP.com [8].

These nanoparticles not only carry peptidoglycan (PG), teichoic acids, and bacterial cytoplasmic components [21], but also selectively enrich key strain-specific effector molecules (Figure 2). Representative examples include cytotoxic molecules from *Bacillus anthracis*, α-hemolysin (Hla) from *Staphylococcus aureus*, hyaluronate lyase from *Cutibacterium acnes*, and listeriolysin O (LLO) from *Listeria monocytogenes* [22].

#### 2.2.2. Biogenesis of Gram-Positive Bacterial MVs

Unlike Gram-negative bacteria, which possess an outer membrane, Gram-positive bacteria lack an outer membrane component in their cell envelope. The formation of MVs in Gram-positive bacteria mainly involves two key biological stages. First, during cytoplasmic membrane budding, dynamic reorganization and enrichment of lipid microdomains occur in specific membrane regions, forming the initial vesicular structures. Second, endogenous lysins degrade the highly cross-linked peptidoglycan (PG) layer through specific hydrolytic activity, thereby regulating cell wall porosity and allowing MVs to pass through the cell wall. This process provides the necessary mechanical support for the final release of MVs [14,23,24].

For example, MV formation in *Staphylococcus aureus* is associated with the production of phenol-soluble modulins (PSMs). PSMs enhance membrane fluidity, while MV production is accompanied by reduced peptidoglycan cross-linking. In addition, *S. aureus* utilizes autolysins to create pores in the cell wall, thereby facilitating MV release [22]. 

## 3. Conditions Affecting BEV Production

### 3.1. Growth Conditions

Temperature affects both the production and functional properties of BEVs, mainly through two mechanisms. First, heat stress can activate bacterial stress-response pathways. For example, *Escherichia coli* produces significantly more BEVs under high-temperature conditions, which may be related to stress signaling triggered by protein denaturation [25]. Second, temperature can influence BEV formation and cargo composition by changing membrane fluidity. In *Staphylococcus aureus*, culture at 40 °C markedly increases the enrichment of virulence factors in vesicles, whereas culture at 34 °C is associated with greater proteomic diversity [26]. One possible explanation is that high temperature may be sensed by *S. aureus* as a host-associated stress cue, particularly under inflammatory or febrile conditions. Under such conditions, selective enrichment of virulence-associated proteins in MVs could help *S. aureus* damage host cells, modulate local immune responses, or remodel the infection microenvironment. In contrast, lower temperatures may favor broader proteomic packaging, which could be more relevant to environmental persistence or colonization outside highly inflammatory niches. Therefore, temperature may influence not only MV yield but also functional cargo selection.

The effect of temperature on BEV yield, however, is not necessarily explained by a single mechanism. When *S. aureus* is cultured to the same optical density at 30 °C, its BEV production is significantly higher than that observed at 37 °C or 40 °C [27]. This suggests that, in addition to membrane fluidity, lower temperatures may also affect BEV release indirectly by altering membrane tension or bacterial energy metabolism.

The composition of the culture medium also influences BEV production by altering bacterial metabolic activity and membrane dynamics. Iron limitation significantly increases vesicle production in *S. aureus* [28], whereas members of the Enterobacteriaceae family respond to nutrient limitation by upregulating OMV biogenesis pathways as part of their adaptation to environmental stress [29].

Antibiotic stress can also promote BEV release through different mechanisms, including interference with peptidoglycan synthesis, disruption of cell envelope integrity, perturbation of membrane homeostasis, and activation of general stress responses. For example, subinhibitory concentrations of tetracycline, ampicillin, and ceftriaxone increase OMV production in *E. coli* by approximately 2- to 4-fold, whereas polymyxin B and colistin can induce an approximately 10-fold increase [30]. Among these antibiotics, ampicillin and ceftriaxone are β-lactam antibiotics that interfere with peptidoglycan synthesis, polymyxin B and colistin are membrane-targeting peptide antibiotics, and tetracycline inhibits bacterial protein synthesis. Therefore, antibiotic-induced BEV production may reflect multiple stress pathways rather than a single cell-wall-targeting mechanism. Similarly, treatment with 64 μg/mL ampicillin increases vesicle production in *S. aureus* by 22.4-fold [31]. This increase is likely linked to antibiotic-induced interference with cell wall synthesis. When peptidoglycan cross-linking is impaired, increased intracellular pressure may drive localized membrane protrusion and accelerate BEV shedding.

### 3.2. Genetic Conditions

BEV biogenesis is finely controlled by multilayered genetic regulatory networks. In *Salmonella*, the outer membrane protein OmpA serves as a key structural protein that anchors the outer membrane to the peptidoglycan layer. Loss of OmpA markedly increases OMV release [32]. However, OMV biogenesis is not governed by a single gene. A genome-wide study in *Escherichia coli* showed that nearly 150 genes are involved in regulating vesicle formation [7].

Further studies suggest that specific regulatory factors may also be important for MV production in Gram-positive bacteria. In *Listeria monocytogenes*, for example, MV production is strongly dependent on the regulatory factor SigB. Comparative culture experiments using the wild-type strain and the Δ*sigB* mutant showed that both strains were able to secrete MVs, but the wild-type strain produced approximately nine times more vesicles than the mutant [33].

The σ^E pathway is a major signaling pathway that monitors and responds to misfolded envelope proteins. It regulates the expression of genes encoding protein-folding and degradation factors, including periplasmic chaperones and proteases, thereby helping bacteria cope with protein misfolding in the periplasm. When the σ^E pathway is impaired, Gram-negative bacteria cannot properly manage the misfolding and aggregation of envelope proteins, leading to the accumulation of potentially harmful materials. Under these conditions, OMV production is upregulated, possibly as a compensatory mechanism to relieve the burden caused by unresolved proteostasis imbalance [29,34].

## 4. BEV-Mediated Bacterial Survival Strategies

### 4.1. BEVs Help Bacteria Resist Environmental Stress

In the hostile environments generated by the host to eliminate pathogenic bacteria, remodeling of membrane lipids and proteins is essential for bacterial survival [9]. The outer membrane of Gram-negative bacteria is structurally asymmetric and heterogeneous, which makes rapid adaptation to environmental changes particularly challenging. BEV secretion may therefore serve as an active route by which bacteria remove unfavorable LPS species from the cell envelope.

In acidic environments, *Salmonella* activates the PhoP/Q and PmrA/B two-component systems. Under these conditions, vesicles selectively export palmitoylated lipid A, while lipid A modified with phosphate groups is retained in the outer membrane. This selective removal of unfavorable LPS helps prevent impairment of membrane stability [35]. Beyond eliminating disadvantageous membrane components, OMVs can also neutralize external threats. For example, exposure to environmental toxins stimulates OMV secretion, and OMVs can adsorb toxic substances to form a physical barrier, thereby reducing direct damage to bacterial cells [36].

OMVs are also involved in biofilm formation, bacterial aggregation, and horizontal gene transfer, all of which improve bacterial tolerance to hostile environments [37,38,39]. Similarly, Gram-positive bacteria use MVs to cope with environmental stress. Under antibiotic pressure, MV production by methicillin-resistant *Staphylococcus aureus* (MRSA) increases by 22.4-fold, and these MVs are enriched in proteins associated with β-lactam antibiotic degradation [31]. These observations suggest that MVs may provide an additional extracellular protective mechanism under antibiotic stress. However, the protective role of MVs should not be interpreted as replacing classical bacterial resistance mechanisms, such as efflux systems, altered antibiotic targets, or enzymatic antibiotic inactivation within bacterial cells. Rather, MVs may provide an additional extracellular layer of protection by carrying β-lactamase-associated proteins and reducing the effective antibiotic pressure in the surrounding microenvironment. In this sense, MV-mediated protection may complement, rather than dominate, conventional antibiotic resistance strategies. In addition, environmental stimuli such as temperature shifts, iron limitation, oxidative stress, alcohol exposure, and salt stress can all trigger increased BEV secretion [27,40].

Thus, BEV secretion represents an important adaptive mechanism by which bacteria maintain homeostasis and survive under adverse conditions. Although BEV biogenesis consumes substantial membrane materials and energy reserves, its net benefit under critical survival pressure may far exceed the associated metabolic cost.

### 4.2. BEVs as Passive Defensive Decoys

As natural derivatives of the bacterial membrane, BEVs share a high degree of compositional similarity with their parental bacteria. This feature has allowed them to develop a distinctive “decoy” function in host–bacteria interactions. When parental bacteria are subjected to targeted attack, vesicles can divert the attack away from the bacterial cell itself and thereby provide protection.

For example, *Escherichia coli* markedly increases OMV secretion when challenged by T4 bacteriophages [41]. Because OMVs carry membrane receptors similar to those on the parental bacterial surface, they can act as decoys that competitively bind bacteriophages in the surrounding environment, preventing phage adsorption onto bacterial cells. OMVs can also function as antibiotic-binding decoys. For instance, OMVs secreted by *E. coli* can bind polymyxin B and protect bacteria from polymyxin B-mediated killing [42].

Vesicles from Gram-positive bacteria play a similar role in evading antibiotic attack. *Staphylococcus aureus* MVs can serve as carriers for the release of the β-lactamase protein BlaZ. These vesicles not only degrade ampicillin in the surrounding environment but also deliver BlaZ to susceptible bacteria through vesicle internalization, enabling them to acquire β-lactamase activity and thereby markedly improving the survival of mixed bacterial populations under antibiotic pressure. In addition, *S. aureus*-derived vesicles can protect bacteria from daptomycin-mediated killing in vitro [43,44].

Together, these examples suggest that BEVs allow bacteria to distribute defensive functions across the population rather than relying solely on individual cellular resistance. By sacrificing a portion of membrane components and energy resources to build a “disposable barrier”, bacteria can preserve the integrity of essential cellular functions while gaining a survival advantage in hostile environments.

### 4.3. BEVs Modulate Host Immune Responses to Support Bacterial Survival

The release of BEVs can activate host immune responses, which may appear disadvantageous for bacterial survival. However, bacteria can balance immune activation and survival needs by adjusting both the amount and the cargo of BEVs. In this way, pathogens may reach a compromise between the infection-promoting functions of BEVs and the risk of immune exposure, thereby facilitating successful colonization of the host.

For example, in complex environments such as the host intestine, *Vibrio cholerae* rapidly adapts by secreting OMVs containing the outer membrane porin OmpT, while avoiding excessive immune activation [45]. In contrast, *Moraxella* appears to use a more active immune-modulating strategy. *Moraxella*-derived OMVs carrying IgD-binding proteins specifically bind to IgD receptors on the surface of host B cells. Through TLR-dependent signaling, these vesicles induce nonspecific immune activation and promote the production of polyclonal IgM antibodies. Importantly, these antibodies are not directed against *Moraxella* antigens themselves, but instead interfere with antigen-specific host immune responses, thereby promoting immune evasion and respiratory tract colonization [46].

The strategy used by *Listeria monocytogenes* further highlights the importance of cargo regulation. During infection of microglia in the central nervous system, *L. monocytogenes* activates the NF-κB/ROS and ERK pathways, triggers assembly of the NLRP3 inflammasome, and induces pyroptosis together with the release of the inflammatory cytokine IL-1β [47]. Faced with such a strong host response, which may also be detrimental to bacterial survival, *L. monocytogenes* appears to have evolved a more subtle strategy. Its secreted MVs act as protective delivery vehicles that encapsulate the key virulence factor listeriolysin O (LLO) and other toxins. This packaging not only protects LLO from degradation by extracellular proteases, but also reduces the risk of immune recognition caused by exposure to free toxins [48].

Taken together, these examples illustrate distinct immune-related survival strategies mediated by BEVs. Vesicles from *V. cholerae* function as tools for environmental adaptation and persistence; *Moraxella* uses OMVs to “hijack” host immune responses for its own benefit; and *L. monocytogenes* MVs serve as concealed delivery vehicles for virulence factors. These strategies reflect the long-term evolutionary interplay between pathogens and hosts.

### 4.4. BEVs Suppress Competing Bacteria

Within the host microenvironment, bacteria can use BEVs to build complex quorum-sensing networks. This form of “microbial language” not only coordinates intraspecies behavior but also shapes the ecological balance of microbial communities. Studies have shown that BEVs can carry quorum-sensing molecules, metabolites, and genetic material, thereby establishing channels for interspecies communication among bacterial populations [6]. Through BEV secretion, bacteria interact with neighboring microorganisms and remodel the local microbial microenvironment [49].

When facing bacterial competitors, *Lactobacillus acidophilus* can secrete MVs that deliver bacteriocins, enabling targeted killing of competing intestinal bacteria [50]. OMVs from *Pseudomonas aeruginosa* can lyse *Staphylococcus aureus*, *Escherichia coli*, and other *Pseudomonas* species [51]. The mode of attack differs according to the structure of the target bacterium. For Gram-positive bacteria, *P. aeruginosa* OMVs attach to the cell wall surface and release hydrolytic enzymes, which locally digest peptidoglycan and lead to bacterial lysis. For Gram-negative bacteria, the outer membrane of *P. aeruginosa* OMVs can fuse with the outer membrane of the target cell, delivering hydrolytic enzymes into the periplasm, where they degrade peptidoglycan and cause cell lysis [52].

Through this target-dependent mode of attack, *P. aeruginosa* can suppress neighboring bacteria with different envelope architectures and strengthen its ecological position within mixed communities. It also illustrates the “ecological dominance” mediated by BEVs and highlights the evolutionary sophistication of BEVs as bacterial weapons.

## 5. Immunomodulatory Effects of BEVs

### 5.1. Interactions Between BEVs and Epithelial Cells

As the first line of host defense against microbial invasion, mucosal surfaces in the vagina, respiratory tract, and intestine are key sites where BEVs initially interact with the host [53,54,55]. At these sites, BEVs carrying diverse biological materials from their parental bacteria can trigger host immune responses [56,57].

Bacterial BEVs first interact with host epithelial cells, leading to cytokine production, altered cell proliferation, apoptosis, and immune activation. For example, stimulation with *Helicobacter pylori* OMVs has been shown to promote gastric epithelial cell proliferation and induce IL-8 production [58]. Vesicles derived from *Escherichia coli* can bind to and be internalized by human intestinal epithelial cells, delivering toxins into the intracellular compartment. The virulence factors released from these vesicles activate the caspase-9-mediated mitochondrial apoptotic pathway, resulting in programmed cell death, and also induce robust IL-8 secretion by intestinal epithelial cells [59].

OMVs from *Moraxella catarrhalis* bind to lipid raft domains in alveolar epithelial cells and are internalized after interacting with TLR2. Once internalized, OMV-associated proteins are delivered into host tissues, where they modulate pro-inflammatory responses in epithelial cells [60]. Similar effects have also been observed for Gram-positive bacteria. MVs derived from *Staphylococcus aureus* can efficiently deliver α-hemolysin into the cytoplasm of epithelial cells, thereby inducing apoptosis [61].

### 5.2. Interactions Between BEVs and Immune Cells

After crossing the epithelial barrier, BEVs can further penetrate into the submucosal layer and interact with various immune cells located beneath the mucosa [62]. Depending on their bacterial origin and virulence-associated cargo, BEVs modulate immune cell functions in different ways.

For example, when polymorphonuclear neutrophils (PMNs) were stimulated in vitro with OMVs derived from *Neisseria meningitidis*, the production of pro-inflammatory cytokines and chemokines was observed, including increased expression of TNF-α, IL-1β, and IL-8. Moreover, when OMVs were combined with IFN-γ, PMNs released higher levels of pro-inflammatory mediators than those induced by OMVs alone [63]. These findings suggest that IFN-γ can enhance the ability of *N. meningitidis* OMVs to induce cytokine and chemokine production in PMNs, thereby amplifying inflammatory responses and contributing to anti-meningococcal immunity.

As antigen-presenting cells (APCs), dendritic cells (DCs) can recognize, internalize, and process exogenous antigens for presentation to T cells, thereby initiating adaptive immune responses [64]. BEVs from various bacterial species have been shown to induce DC maturation and cytokine production. OMVs from *Salmonella typhimurium* strongly activate professional APCs in vitro, to a level comparable to that induced by the bacteria themselves. In macrophages and DCs stimulated with these OMVs, the surface expression of MHC-II and CD86 is increased, accompanied by markedly enhanced production of the pro-inflammatory mediators NO, TNF-α, and IL-12 [65]. Similarly, MVs derived from *Listeria monocytogenes* promote the proliferation of murine DCs and macrophages in vitro, and increase the expression of TNF-α, IL-1β, IL-6, and IL-10 in macrophages [66].

### 5.3. BEVs as Bridges from Innate Immunity to Adaptive Immunity

**Figure 3:**
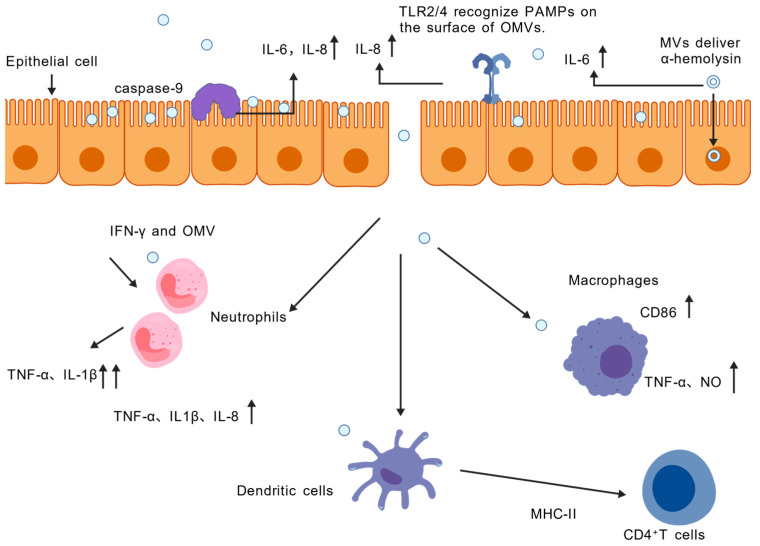
Immunomodulatory effects of bacterial extracellular vesicles (BEVs). The figure was created using BioGDP.com [8].

Although BEVs can activate host immune responses, this process does not necessarily represent a disadvantage for bacteria. Instead, BEVs allow bacteria to shape host immunity in a spatially and temporally controlled manner, thereby creating conditions that may favor bacterial survival, colonization, or immune evasion. By delivering PAMPs and pathogen-associated antigens to host cells, BEVs can initiate early inflammatory signaling, promote epithelial cell activation, stimulate the secretion of pro-inflammatory cytokines, and activate antigen-presenting cells such as macrophages and dendritic cells [15,62,65] (Figure 3). These responses may appear to support host defense; however, from the bacterial perspective, vesicle-mediated immune stimulation can also serve as a strategy to remodel the local immune microenvironment before or during bacterial invasion.

BEVs can further influence the transition from innate to adaptive immunity. As professional antigen-presenting cells, dendritic cells can recognize, internalize, and process BEV-associated antigens for presentation to T cells, thereby initiating adaptive immune responses [61]. For example, OMVs from *Salmonella typhimurium* potently activate macrophages and dendritic cells, upregulate MHC-II and CD86 expression, and stimulate the production of NO, TNF-α, and IL-12 [65]. Although these responses contribute to protective immunity, they may also help bacteria distribute antigens at a distance from intact bacterial cells, allowing pathogens to divert immune recognition toward vesicle-associated components rather than the parental bacteria themselves. In this sense, BEVs may function as immunological decoys that expose selected bacterial antigens while preserving the viability and integrity of bacterial cells.

Some bacteria use BEVs more directly to reshape adaptive immune responses in their favor. OMVs from *Moraxella catarrhalis* can interact with B cells through IgD-binding proteins and induce TLR-dependent polyclonal B-cell activation, resulting in nonspecific IgM production [46]. This response may interfere with antigen-specific immunity and thereby promote immune evasion and respiratory tract colonization. Thus, BEVs can bridge innate and adaptive immunity not only by promoting antigen presentation, but also by redirecting or diluting host immune responses in ways that benefit bacterial persistence.

From the perspective of host defense, BEV secretion appears to present a clear evolutionary paradox. The PAMPs carried by BEVs can be recognized by host PRRs and induce immune responses aimed at pathogen clearance, which should theoretically impose negative pressure on pathogenic bacteria [11,15]. However, bacteria appear to exploit this risk by using BEVs as flexible immunomodulatory tools. Through selective cargo packaging, long-distance antigen delivery, inflammatory microenvironment remodeling, and immune decoy effects, BEVs allow bacteria to tolerate, reshape, or evade host immunity [9]. Therefore, BEVs should be viewed as bidirectional mediators: they can alert the host immune system, but they can also provide bacteria with strategic advantages depending on the bacterial species, vesicle cargoes, host niche, and immune context.

### 5.4. BEVs as Mediators of Host–Commensal–Pathogen Interactions

In addition to vesicles released by classical pathogens, BEVs from anaerobic commensals and health-promoting bacteria have attracted increasing interest. This is particularly relevant in mucosal niches, where host cells, commensals, probiotics, and invading pathogens coexist in the same ecological space [67,68]. In such environments, BEVs are not merely carriers of virulence factors. They can also act as mobile packages of bacterial information, allowing different members of the microbial community to communicate with host cells and with each other [49]. Representative examples of BEVs involved in host–commensal–pathogen interactions are summarized in Table 1.

Anaerobic bacteria are an important source of BEVs, although they have been less extensively discussed than aerobic or facultative anaerobic pathogens. For example, *Bacteroides fragilis*, a major anaerobic commensal in the gut, releases OMVs enriched in polysaccharide A. These vesicles can interact with dendritic cells through TLR2-dependent signaling, promote regulatory T-cell responses, and induce IL-10 production, thereby contributing to intestinal immune tolerance and protection against experimental colitis [69]. In addition, *B. fragilis* OMVs contain proteins, LPS, peptidoglycan, DNA, and RNA, which may explain why they can engage multiple innate immune receptors more broadly than the parental bacterium itself [70]. This example shows that anaerobic commensal-derived BEVs are not simply inflammatory stimuli; under certain conditions, they may help preserve mucosal immune balance. 

By contrast, BEVs from some anaerobic bacteria are more closely associated with pathogenic processes. *Porphyromonas gingivalis*, a Gram-negative obligate anaerobe involved in periodontitis, releases OMVs containing gingipains, fimbriae, LPS, outer membrane proteins, and small RNAs. These vesicles can promote epithelial inflammation, immune modulation, biofilm development, and polymicrobial pathogenicity in the oral cavity [71,72,73]. Their effects are also strongly shaped by the surrounding microbial community. Therefore, *P. gingivalis* OMVs should not be viewed only as isolated virulence carriers, but also as mediators that help organize oral biofilm ecology and influence interactions between pathogens and commensals. 

BEVs from health-promoting bacteria add another layer of complexity. *Lactobacillus acidophilus* MVs can deliver bacteriocins and inhibit competing intestinal bacteria, suggesting that probiotic-derived vesicles may participate in microbial community control [50]. Akkermansia muciniphila, a mucin-degrading anaerobic commensal, produces EVs that improve intestinal barrier integrity by increasing tight-junction protein expression and regulating gut permeability in experimental models [67,74]. These examples suggest that some beneficial effects attributed to probiotic or commensal bacteria may, at least in part, be mediated by their vesicles.

Overall, the function of BEVs depends heavily on the ecological and immunological context. In the intestine, oral cavity, respiratory tract, and skin, BEVs may support commensal colonization, restrict competing microbes, modulate host immunity, or facilitate pathogen persistence [67]. Thus, BEVs are best understood not only as pathogen-derived virulence tools but also as active participants in host-associated microbial communities, where they shape the balance among host defense, commensal stability, and pathogen invasion.

**Table 1:** Representative bacterial extracellular vesicles: characterization methods, size ranges, major cargoes, and biological functions.

Bacterial Species	Gram Type/Oxygen Relationship	EV Type	Reported Size Range	Common Characterization Methods	Major Components/Cargoes	Representative Biological Functions	References
*Escherichia coli*	Gram-negative, facultative anaerobe	OMVs	Approximately 20–250 nm	TEM, cryo-EM, NTA, protein quantification, proteomics, LPS analysis	LPS, OmpA, OmpC, GroEL, periplasmic proteins, DNA/RNA, toxins in pathogenic strains	Stress adaptation, toxin delivery, epithelial injury, cytokine induction, antimicrobial resistance transfer	[5,10,29,30,59]
*Salmonella enterica* serovar Typhimurium	Gram-negative, facultative anaerobe	OMVs	Approximately 50–250 nm	TEM, NTA, immunoblotting, proteomics, cytokine assays	LPS, outer membrane proteins, peptidoglycan fragments, DNA, immunogenic antigens	DC and macrophage activation, MHC-II/CD86 upregulation, NO, TNF-α and IL-12 induction	[35,65]
*Vibrio cholerae*	Gram-negative, facultative anaerobe	OMVs	Approximately 50–250 nm	TEM, NTA, lipidomics, proteomics, infection models	LPS, OmpT, outer membrane proteins, virulence-associated proteins	Surface remodeling, intestinal adaptation, host colonization	[45]
*Pseudomonas aeruginosa*	Gram-negative, aerobic	OMVs	Approximately 50–300 nm	TEM, NTA, proteomics, bacterial killing assays	LPS, quorum-sensing molecules, hydrolytic enzymes, virulence factors	Biofilm formation, interbacterial killing, delivery of peptidoglycan-degrading enzymes	[51,52]
*Bacteroides fragilis*	Gram-negative, obligate anaerobe	OMVs	Approximately 50–250 nm	TEM, NTA, proteomics, nucleic acid detection, epithelial/immune cell assays	Polysaccharide A, LPS, peptidoglycan, proteins, DNA and RNA	Immune tolerance, TLR2-dependent Treg induction, IL-10 production, protection against colitis	[68,69,70]
*Porphyromonas gingivalis*	Gram-negative, obligate anaerobe	OMVs	Approximately 50–300 nm	TEM, NTA, proteomics, enzymatic activity assays, epithelial cell assays	Gingipains, fimbriae, LPS, outer membrane proteins, nucleic acids	Periodontal inflammation, immune modulation, biofilm development, polymicrobial pathogenicity	[71,72]
*Staphylococcus aureus*	Gram-positive, facultative anaerobe	MVs	Approximately 20–300 nm	TEM, NTA, SDS-PAGE, proteomics, toxin assays	Peptidoglycan, lipoteichoic acid, α-hemolysin, β-lactamase, cytoplasmic proteins	Epithelial apoptosis, antibiotic defense, inflammatory activation, toxin delivery	[26,27,28,31,43,44,61]
*Listeria monocytogenes*	Gram-positive, facultative anaerobe	MVs	Approximately 50–300 nm	TEM, NTA, proteomics, immunoblotting, macrophage/DC assays	LLO, internalin-related proteins, lipoproteins, peptidoglycan, teichoic acids	Virulence factor delivery, macrophage activation, inflammatory signaling, host–cell modulation	[33,48,66]
*Lactobacillus acidophilus*	Gram-positive, aerotolerant anaerobe	MVs	Approximately 20–200 nm	TEM, NTA, proteomics, antimicrobial assays	Bacteriocins, lipoteichoic acid, membrane proteins, enzymes	Inhibition of competing bacteria, host immune modulation, probiotic/postbiotic effects	[50]
*Akkermansia muciniphila*	Gram-negative, anaerobic mucin-degrading commensal	OMVs/EVs	Approximately 50–200 nm	TEM, NTA, epithelial barrier assays, animal models	Outer membrane proteins, lipids, enzymes, immunomodulatory cargoes	Tight-junction regulation, gut barrier protection, metabolic and inflammatory modulation	[74]

## 6. Conclusions and Perspectives

Accumulating evidence indicates that BEV production can provide important advantages for bacteria under specific environmental and host-associated conditions. BEVs from both Gram-negative and Gram-positive bacteria carry specific molecular components. Although vesicle secretion requires considerable energy investment and biological cost, BEVs provide bacteria with a rapid and selective mechanism for membrane remodeling, thereby supporting bacterial survival under changing environmental conditions.

Bacterial BEVs may be viewed as one of the most refined products of microbial adaptation. They function not only as a “nanoscale arsenal” in pathogen offensive and defensive strategies, but also as molecular messengers in host–microbe co-evolution. On the one hand, BEVs can protect parental bacteria under antibiotic pressure by acting as decoys. A deeper understanding of key BEV-associated proteins may support the development of new antibacterial strategies that inhibit BEV secretion, thereby blocking virulence factor delivery and immune evasion. In addition, the antibacterial activity displayed by BEVs during interspecies competition offers a potential intervention strategy for infections caused by drug-resistant bacteria. On the other hand, the broader significance of BEVs lies in their potential as natural immune activators. Because they can induce strong and durable immune responses in vivo, BEVs may provide useful guidance for the design of vesicle-based vaccines.

However, for this field to move from proof-of-concept studies toward clinical translation, safety remains the primary challenge. Although BEVs are potent immune stimulators, they may also retain pathogen-associated virulence factors, raising concerns about excessive inflammation or potential infection-related risks. Two major strategies may help address this problem. At the molecular design level, genetic engineering can be used to modify parental bacterial strains. For example, deletion of the gene encoding the *Listeria monocytogenes* virulence factor listeriolysin O (LLO) may allow the production of attenuated but immunogenic engineered MVs that retain PAMPs capable of activating M1-like macrophage polarization. At the translational level, rigorous in vivo studies are needed to comprehensively evaluate the immune effects and toxicity profiles of BEVs under different doses and administration routes in relevant animal models, so as to identify an optimal therapeutic window with acceptable safety and efficacy.

Taken together, two major directions—vesicle-targeted antibacterial therapy and vesicle-based vaccine design—may represent a shift from passive response to active intervention in the evolutionary contest between humans and pathogens. Elucidating the roles of BEVs in infection and immunity will not only improve strategies for pathogen prevention and control but may also open new possibilities in fields such as cancer immunotherapy. Further investigation into the biological mechanisms of BEVs will be essential for turning these bacterial products from survival tools of pathogens into innovative platforms for biomedical application.

## Data Availability

No new data were created or analyzed in this study. Data sharing is not applicable to this article.

## Abbreviations

The following abbreviations are used in this manuscript:

BEVs	Bacterial extracellular vesicles
OMVs	Outer membrane vesicles
MVs	Membrane vesicles
LPS	Lipopolysaccharide
PG	Peptidoglycan
PAMPs	Pathogen-associated molecular patterns
PRRs	Pattern recognition receptors
TLRs	Toll-like receptors
DCs	Dendritic cells
PMNs	Polymorphonuclear neutrophils
MRSA	Methicillin-resistant *Staphylococcus aureus*
LLO	Listeriolysin O
